# First report of two Eriophyid mite genera (Acariformes, Eriophyidae) from Saudi Arabia with description of a new species and a key to known *Cosetacus* species

**DOI:** 10.3897/BDJ.14.e194689

**Published:** 2026-05-20

**Authors:** Eid Muhammad Khan, Jawwad Hassan Mirza, Fahad Jaber Alatawi

**Affiliations:** 1 Department of Plant Protection, College of Food and Agriculture Sciences, King Saud University, Riyadh, Saudi Arabia Department of Plant Protection, College of Food and Agriculture Sciences, King Saud University Riyadh Saudi Arabia

**Keywords:** genera records, key, morphology, new species, taxonomy

## Abstract

**Background:**

Two eriophyoid mite genera, *Cosetacus* Keifer and *Neserella* Meyer & Ueckermann, are reported for the first time in Saudi Arabia (SA). A new species, *Cosetacus javanicus* **sp. nov**., is described and illustrated, based on the female specimens. Two species, *Cecidophyopsis vermiformis* (Nalepa) and *Neserella capreifoliae* Meyer & Ueckermann, are newly recorded for the eriophyid mite fauna of SA. In addition, a key to known species of *Cosetacus* is provided.

**New information:**

Two eriophyid mite genera, *Cosetacus* Keifer and *Neserella* Meyer & Ueckermann (Acariformes, Eriophyidae, Cecidophyinae) are herein recorded for the first time from Saudi Arabia (SA), with a new species, *C. javanicus* **sp. nov**., collected from *Aerva javanica* (Burm. f.) (Amaranthaceae). Moreover, two species, *Cecidophyopsis vermiformis* (Nalepa) collected from *Conocarpus erectus* (Combretaceae R. Br.), and *Neserella capreifoliae* Meyer & Ueckermann collected from *Ficus carica* (Moraceae Link), are reported as new records for the eriophyoid mite fauna of SA. The individuals of these species are vagrants on the abaxial leaf surface, causing no apparent symptoms to the host plant species. Additionally, a key to known species of *Cosetacus* is provided.

## Introduction

The two eriophyid genera, *Cosetacus* Keifer and *Neserella* Meyer & Ueckermann (Eriophyidae, Cecidophyinae) are poorly known genera in the tribes Colomerini and Cecidophyini, respectively (Meyer and Ueckermann 1989, Amrine et al. 2003). The genus *Cosetacus* was erected, based on *Aceria camelliae* (Keifer) and diagnosed by the lack of paraxial-tibial setae. It includes six species mainly recorded from Australia, Brazil, China, Spain, Korea, India and New Zealand (Keifer 1966, Das and Chakrabarti 1985, Chakrabarti and Pandit 1997, Pandit and Chakrabarti 2007, Menon et al. 2009, Han and Zhang 2019).

The genus *Neserella* was erected, based on *N. decora* Meyer & Ueckermann, which included two additional species, *N. trema* Meyer & Ueckermann and *N. capreifoliae* Meyer & Ueckermann. It is morphologically very close to *Cecidophyopsis* Keifer and differentiated by the absence of anterolateral setae on coxisternum I (1*b*), second pair of opisthosomal seta (*e*) and paraxial-tibial seta (*l’*) (Amrine et al. 2003). The genus *Milleniophyes* Chandrapatya & Boczek, which was subsequently synonymised with *Neserella* and its type species considered a junior synonym of *N. capreifoliae* (Chandrapatya et al. 2016). Recently, *N. capreifoliae* was recorded on *Ficus carica* L. (Moraceae) from Egypt and supplementary line drawings were provided (Elhalawany et al. 2022).

Previously, two genera and three species of the subfamily Cecidophyinae were known from Saudi Arabia (Al-Atawi and Halawa 2011, Wang et al. 2014). In the present study, two genera, *Cosetacus* and *Neserella*, are recorded for the first time, with a new species, *C. javanicus* sp. nov. and two species records, *Cecidophyopsis vermiformis* (Nalepa) and *N. capreifoliae*. Additionally, a key to the known species of the genus *Cosetacus* is provided.

## Materials and methods

Plant foliage (leaves and twigs) was snipped and placed in field-labelled polythene bags along with paper towels and stored in an ice box. Mite specimens were recovered by the modified washing method following (Monfreda et al. 2007). The collected specimens were cleared in lactic acid at room temperature and mounted in Keifer F-medium (Amrine and Manson 1996), as updated by de Lillo et al. (2010). The morphological terminology and setal notation follow Lindquist (1996). The genus of the new species and new record was identified by following the generic key of Amrine et al. (2003) and the genera erected since 2003 in the respective tribe. Different body parts were imaged using auto-montage software (Syncroscopy, Cambridge, UK) attached to the compound microscope (DM2500, Leica®, Germany). Some body parts were hand-drawn with a drawing tube Camera Lucida attached to the Olympus microscope (BX51, Olympus®, Japan). These images were used as a template for the schematic line drawings, drawn with Adobe Illustrator (Adobe Systems Inc., San Jose, CA, USA). The morphological traits were measured by following Lindquist (1996), as modified by de Lillo et al. (2010). All measurements are given in micrometres (μm). The holotype's measurements are followed by the range values of the paratypes in parentheses. The measurements were rounded off to the nearest integer and regarded as the length of the morphological traits unless otherwise specified. The plant samples were identified from the Department of Botany at the College of Science, King Saud University (KSU). All specimens of new species and new records have been deposited in the King Saud University Museum of Arthropods (KSMA, Acarology section), Department of Plant Protection, College of Food and Agriculture Sciences, King Saud University, Riyadh, Saudi Arabia.

## Taxon treatments

### Cosetacus javanicus sp. nov.

0AD2E577-072A-5EDD-9471-C85F6AFA693B

3411DAD5-BA81-4D6D-882A-EE03EBA87724

#### Materials

- **Type status:** Holotype. **Occurrence:** catalogNumber: KSMAAS-22-Eri-Cos-H; recordedBy: E.M. Khan; individualCount: 1; sex: Female; lifeStage: adult; occurrenceID: B042D76B-46C7-51C9-A0FA-6653353E165A; **Taxon:** scientificName: Cosetacus javanicus ; kingdom: Animalia; phylum: Arthropoda; class: Arachnida; order: Prostigmata; family: Eriophyidae; genus: Cosetacus; **Location:** country: Saudi Arabia; stateProvince: Abha; verbatimCoordinates: 18°9'25.2''N, 42°30'32.5''E; decimalLatitude: 18.157; decimalLongitude: 42.50902; georeferenceProtocol: GPS; **Identification:** identifiedBy: Eid Muhammad Khan; dateIdentified: 2022; **Event:** samplingProtocol: Leaves collection; eventDate: 11/6/2022; habitat: Aerva javanica ; **Record Level:** language: en; collectionCode: Mites; basisOfRecord: Slide Mounted Specimen
- **Type status:** Paratype. **Occurrence:** catalogNumber: KSMAAS-22-Eri-Cos-P1; recordedBy: E.M. Khan; individualCount: 1; sex: Female; lifeStage: adult; occurrenceID: D2E06FA0-A198-5F91-B9FC-61D60B606A5E; **Taxon:** scientificName: Cosetacus javanicus ; kingdom: Animalia; phylum: Arthropoda; class: Arachnida; order: Prostigmata; family: Eriophyidae; genus: Cosetacus; **Location:** country: Saudi Arabia; stateProvince: Abha; verbatimCoordinates: 18°9'25.2''N, 42°30'32.5''E; decimalLatitude: 18.157; decimalLongitude: 42.50902; georeferenceProtocol: GPS; **Identification:** identifiedBy: Eid Muhammad Khan; dateIdentified: 2022; **Event:** samplingProtocol: Leaves collection; eventDate: 11/6/2022; habitat: Aerva javanica ; **Record Level:** language: en; collectionCode: Mites; basisOfRecord: Slide Mounted Specimen
- **Type status:** Paratype. **Occurrence:** catalogNumber: KSMAAS-25-Eri-Cos-P2; recordedBy: E.M. Khan; individualCount: 1; sex: Female; lifeStage: adult; occurrenceID: 7FFBF157-3409-5EB5-83EC-4356627D5B08; **Taxon:** scientificName: Cosetacus javanicus ; kingdom: Animalia; phylum: Arthropoda; class: Arachnida; order: Prostigmata; family: Eriophyidae; genus: Cosetacus; **Location:** country: Saudi Arabia; stateProvince: Jazan; verbatimCoordinates: 17°20'14.435"N 42°33'9.197"E; decimalLatitude: 17.337; decimalLongitude: 42.55255; georeferenceProtocol: GPS; **Identification:** identifiedBy: Eid Muhammad Khan; dateIdentified: 2025; **Event:** samplingProtocol: Leaves collection; eventDate: 24/02/2025; habitat: Aerva javanica ; **Record Level:** language: en; collectionCode: Mites; basisOfRecord: Slide Mounted Specimen
- **Type status:** Paratype. **Occurrence:** catalogNumber: KSMAAS-25-Eri-Cos-P3; recordedBy: E.M. Khan; individualCount: 1; sex: Female; lifeStage: adult; occurrenceID: 97F90742-814D-5D3E-BA7D-944FC9C70430; **Taxon:** scientificName: Cosetacus javanicus ; kingdom: Animalia; phylum: Arthropoda; class: Arachnida; order: Prostigmata; family: Eriophyidae; genus: Cosetacus; **Location:** country: Saudi Arabia; stateProvince: Jazan; verbatimCoordinates: 17°20'14.435"N 42°33'9.197"E; decimalLatitude: 17.337; decimalLongitude: 42.55255; georeferenceProtocol: GPS; **Identification:** identifiedBy: Eid Muhammad Khan; dateIdentified: 2025; **Event:** samplingProtocol: Leaves collection; eventDate: 24/02/2025; habitat: Aerva javanica ; **Record Level:** language: en; collectionCode: Mites; basisOfRecord: Slide Mounted Specimen
- **Type status:** Paratype. **Occurrence:** catalogNumber: KSMAAS-25-Eri-Cos-P4; recordedBy: E.M. Khan; individualCount: 1; sex: Female; lifeStage: adult; occurrenceID: 95DB0E50-35FB-588E-8AF1-A269BD735092; **Taxon:** scientificName: Cosetacus javanicus ; kingdom: Animalia; phylum: Arthropoda; class: Arachnida; order: Prostigmata; family: Eriophyidae; genus: Cosetacus; **Location:** country: Saudi Arabia; stateProvince: Jazan; verbatimCoordinates: 17°20'14.435"N 42°33'9.197"E; decimalLatitude: 17.337; decimalLongitude: 42.55255; georeferenceProtocol: GPS; **Identification:** identifiedBy: Eid Muhammad Khan; dateIdentified: 2025; **Event:** samplingProtocol: Leaves collection; eventDate: 24/02/2025; habitat: Aerva javanica; **Record Level:** language: en; collectionCode: Mites; basisOfRecord: Slide Mounted Specimen

#### Description

**Figure 1.**
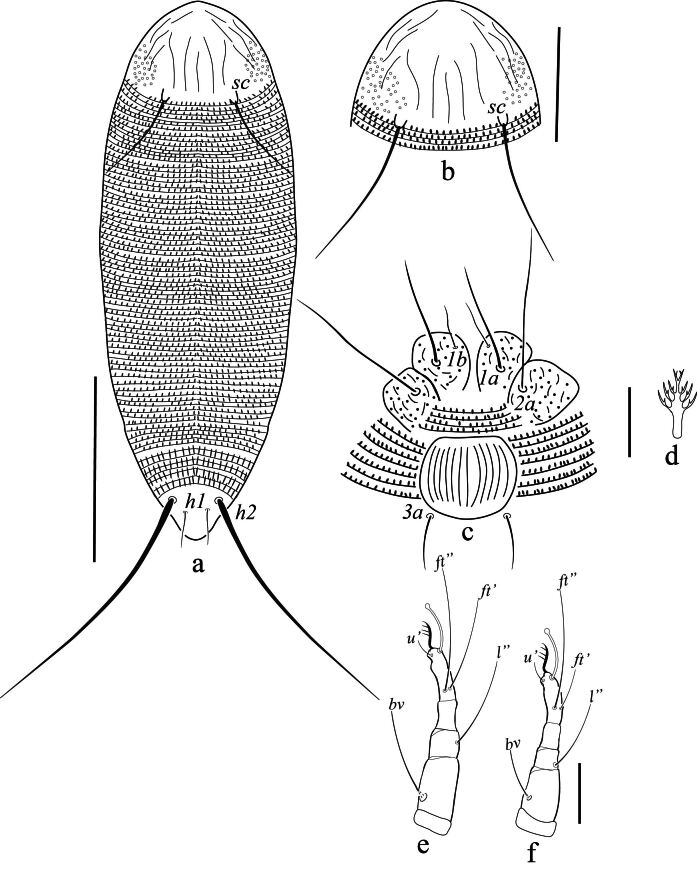
*Cosetacus javanicus* sp. nov. Female: **a** Dorsal view, Scale bar 50 µm; **b** Prodorsal shield, Scale bar 20 µm; **d** Empodium; **c** Coxigenital area, Scale bar 5 µm; **e** Leg І; **f** Leg II, Scale bar 10 µm.

**Figure 2.**
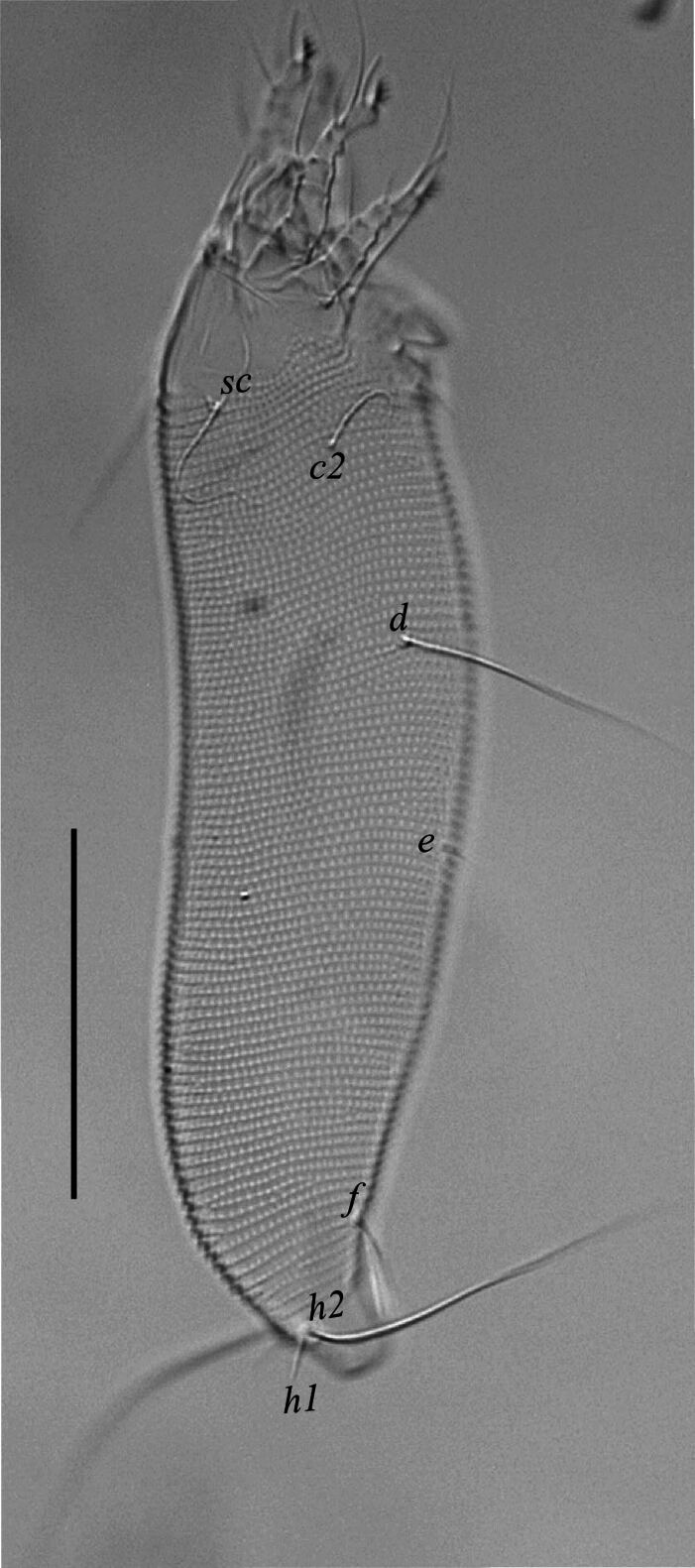
*Cosetacus javanicus* sp. nov. Female, dorso-lateral view. Scale bar 50 µm.

Female (n = 5). Body vermiform (Fig. 1a), 147 (142–155), excluding gnathosoma, 49 (51–54) wide, 44 (40–50) thick. Gnathosoma 16 (15–17), projecting obliquely downwards, cheliceral stylets 15 (13–17) long, palp coxal seta (*ep*) 3 (2–3), simple; dorsal pedipalp genu seta (*d*) 7 (6–8), unfurcated, palp tarsal ventral seta (*v*) indistinct. Prodorsal shield sub oval, 24 (23–25) long, 48 (46–50) wide; prodorsal shield with median line 1/3 of prodorsal shield, and admedian line at posterior half of shield, slightly curving near the middle, first submedian line beginning slightly ahead of scapular tubercle extending to half of the prodorsal shield, others sublateral lines broken and irregular, prodorsal shield laterally with sparse granules, scapular tubercles and setae (*sc*) near the rear shield margin, basal axes transverse, scapular setae (*sc*) 26 (24–28) long, 17 (16–18) apart, projecting posteriorly (Fig. 1b). Coxisternal plates with granules and short lines, sternal line lacking, 4–5 semi-annuli between coxal plate and coxigenital region, anterolateral setae on coxisternum I (1*b*) 7 (6–7) long, 12 (11–13) apart; proximal setae on coxisternum I (1*a*) 20 (19–22) long, 7 (6–7) apart; proximal setae on coxisternum II (2*a*) 35 (32–36) long, 20 (19–22) apart (Fig. 1c). Legs with all usual segments and lacking paraxial-tibial seta. Leg I 27 (25–28), trochanter 3 (2–3), femur 7 (6–8), genu 3 (4–5), tibia 5 (5–6), tarsus 7 (6–8), tarsal empodium (*em*) simple, 4 (4–5), 4-rayed (Fig. 1d), tarsal solenidion (ω) 8 (7–8), distally with large spherical knob; basiventral femoral setae (*bv*) 10 (9–11); antaxial genual setae (*l''*) 17 (16–19); paraxial tibial seta (*l*’) absent; paraxial fastigial tarsal setae (*ft'*) 15 (14–16), antaxial fastigial tarsal setae (*ft''*) 21 (19–21), paraxial unguinal tarsal setae (*u'*) 3 (3–4) (Fig. 1e). Leg II 23 (22–25), trochanter 3 (2–4), femur 8 (7–9), genu 4 (4–5), tibia 4 (4–5), tarsus 7 (6–7), tarsal solenidion (*ω*) 9 (8–10) distally knobbed, tarsal empodium simple, 5 (5–6), 4–rayed (Fig. 1f); basiventral femoral setae (*bv*) 8 (7–10); antaxial genual setae (*l''*) 12 (11–14); paraxial fastigial tarsal setae (*ft'*) 6 (5–6), antaxial fastigial tarsal setae (*ft''*) 21 (19–23), paraxial unguinal tarsal setae (*u'*) 3 (2–3). Opisthosoma dorsal semi-annuli 78 (74–80), continuous dorsoventrally, oval microtubercles, those on posterior 12–15 annuli more elongate and triangular, ventral annuli 66 (63–68), small oval microtubercles, Lateral setae (*c_2_*) 24 (22–24), on ventral semiannulus 10 from coxae II, 42 (40–44) apart; setae (*d*) 45 (43–49), on semi-annulus 26 (25–28), 42 (40–45) apart; setae (*e*) 7 (6–9), on semi-annulus 40 (38–42), 18 (16–20) apart; setae (*f* ) 21 (20–23), on the 6^th^ ventral semiannulus from rear, 16 (15–17) apart; setae (*h_2_*) 78 (75–83); setae (*h_1_*) 10 (9–12) (Fig. 2). Female external genitalia 15 (13–16), 19 (17–21) wide, cover-flap with 10 (9–12) longitudinal striae, proximal setae (3*a*) 10 (9–12), 15 (13–17) apart (Fig. 1c).

**Male**. Not found.

**Relation to the host plant**. Vagrant on the abaxial surface of the leaf. No apparent symptoms to the host foliage were observed.

#### Diagnosis

Scapular tubercles and setae (*sc*) near the rear shield margin, prodorsal shield laterally coarsely granulated, median line at about 1/3 of prodorsal shield, admedian lines at posterior half of prodorsal shield, first submedial line beginning slightly ahead of scapular tubercle extending to half of the prodorsal shield, coxisternal plates with granules and short lines, feather claws 4-rayed and accessory setae (*h_1_*) present.

##### Remarks

*Cosetacus javanicus* sp. nov. is morphologically close to *C. prosteti* Pandit & Chakrabarti and *C. camelliae* (Keifer) by having the characters of prodorsal shield median lines, entire empodium and granules laterally on the prodorsal shield. However, the new species differs from *C. prosteti* (Keifer) by the ornamentation on coxisternal plates, with granules and short irregular lines vs. without ornamentation in *C. prosteti*. Moreover, the new species differs from *C. camelliae* (Keifer) by the incomplete admedian lines vs. complete admedian line in *C. camelliae* and accessory setae (*h_1_*) present vs. accessory setae absent in *C. camelliae* (Keifer).

#### Etymology

The specific epithet is derived from the host plant species “*javanica*” from which the specimens were collected.

### Cecidophyopsis vermiformis

(Nalepa)

166D078F-2BF3-52BA-8600-C361B22BEAEF

#### Materials

- **Type status:** Other material. **Occurrence:** catalogNumber: KSMAAS-21-Eri-Cec-01; recordedBy: E.M. Khan; individualCount: 1; sex: Female; lifeStage: adult; occurrenceID: 4713D08D-20B7-5790-915B-914DD3703E41; **Taxon:** scientificName: Cecidophyopsis vermiformis ; kingdom: Animalia; phylum: Arthropoda; class: Arachnida; order: Prostigmata; family: Eriophyidae; genus: Cecidophyopsis; **Location:** country: Saudi Arabia; stateProvince: Abha; verbatimCoordinates: 19°60'2.67"N 41°32'9.33"E; decimalLatitude: 20.000742; decimalLongitude: 41.535925; georeferenceProtocol: GPS; **Identification:** identifiedBy: Eid Muhammad Khan; dateIdentified: 2021; **Event:** samplingProtocol: Leaves collection; eventDate: 13/09/2021; habitat: Conocarpus erectus ; **Record Level:** language: en; collectionCode: Mites; basisOfRecord: Slide Mounted Specimen
- **Type status:** Other material. **Occurrence:** catalogNumber: KSMAAS-21-Eri-Cec-02; recordedBy: E.M. Khan; individualCount: 1; sex: Female; lifeStage: adult; occurrenceID: 47382F39-228E-5F66-9691-ADDBCE92E13E; **Taxon:** scientificName: Cecidophyopsis vermiformis ; kingdom: Animalia; phylum: Arthropoda; class: Arachnida; order: Prostigmata; family: Eriophyidae; genus: Cecidophyopsis; **Location:** country: Saudi Arabia; stateProvince: Abha; verbatimCoordinates: 19°60'2.67"N 41°32'9.33"E; decimalLatitude: 20.000742; decimalLongitude: 41.535925; georeferenceProtocol: GPS; **Identification:** identifiedBy: Eid Muhammad Khan; dateIdentified: 2021; **Event:** samplingProtocol: Leaves collection; eventDate: 13//09/2021; habitat: Conocarpus erectus ; **Record Level:** language: en; collectionCode: Mites; basisOfRecord: Slide Mounted Specimen
- **Type status:** Other material. **Occurrence:** catalogNumber: KSMAAS-21-Eri-Cec-03; recordedBy: E.M. Khan; individualCount: 1; sex: Female; lifeStage: adult; occurrenceID: FDEDC13D-CD89-578B-8525-DCAE4728C7C2; **Taxon:** scientificName: Cecidophyopsis vermiformis ; kingdom: Animalia; phylum: Arthropoda; class: Arachnida; order: Prostigmata; family: Eriophyidae; genus: Cecidophyopsis; **Location:** country: Saudi Arabia; stateProvince: Abha; verbatimCoordinates: 19°60'2.67"N 41°32'9.33"E; decimalLatitude: 20.000742; decimalLongitude: 41.535925; georeferenceProtocol: GPS; **Identification:** identifiedBy: Eid Muhammad Khan; dateIdentified: 2021; **Event:** samplingProtocol: Leaves collection; eventDate: 13/09/2021; habitat: Conocarpus erectus ; **Record Level:** language: en; collectionCode: Mites; basisOfRecord: Slide Mounted Specimen
- **Type status:** Other material. **Occurrence:** catalogNumber: KSMAAS-21-Eri-Cec-04; recordedBy: E.M. Khan; individualCount: 1; sex: Female; lifeStage: adult; occurrenceID: 80018564-624D-58E2-8B24-B0D3E5BB6FB6; **Taxon:** scientificName: Cecidophyopsis vermiformis ; kingdom: Animalia; phylum: Arthropoda; class: Arachnida; order: Prostigmata; family: Eriophyidae; genus: Cecidophyopsis; **Location:** country: Saudi Arabia; stateProvince: Abha; verbatimCoordinates: 19°60'2.67"N 41°32'9.33"E; decimalLatitude: 20.000742; decimalLongitude: 41.535925; georeferenceProtocol: GPS; **Identification:** identifiedBy: Eid Muhammad Khan; dateIdentified: 2021; **Event:** samplingProtocol: Leaves collection; eventDate: 13/09/2021; habitat: Conocarpus erectus ; **Record Level:** language: en; collectionCode: Mites; basisOfRecord: Slide Mounted Specimen
- **Type status:** Other material. **Occurrence:** catalogNumber: KSMAAS-21-Eri-Cec-05; recordedBy: E.M. Khan; individualCount: 1; sex: Female; lifeStage: adult; occurrenceID: 4BD53F37-FCF8-5AB0-8C31-E1F5F4863E0F; **Taxon:** scientificName: Cecidophyopsis vermiformis ; kingdom: Animalia; phylum: Arthropoda; class: Arachnida; order: Prostigmata; family: Eriophyidae; genus: Cecidophyopsis; **Location:** country: Saudi Arabia; stateProvince: Abha; verbatimCoordinates: 19°60'2.67"N 41°32'9.33"E; decimalLatitude: 20.000742; decimalLongitude: 41.535925; georeferenceProtocol: GPS; **Identification:** identifiedBy: Eid Muhammad Khan; dateIdentified: 2021; **Event:** samplingProtocol: Leaves collection; eventDate: 13/09/2021; habitat: Conocarpus erectus ; **Record Level:** language: en; collectionCode: Mites; basisOfRecord: Slide Mounted Specimen
- **Type status:** Other material. **Occurrence:** catalogNumber: KSMAAS-21-Eri-Cec-06; recordedBy: E.M. Khan; individualCount: 1; sex: Female; lifeStage: adult; occurrenceID: 5823DC34-2AC9-5EC3-B6F3-8C9350C919C6; **Taxon:** scientificName: Cecidophyopsis vermiformis ; kingdom: Animalia; phylum: Arthropoda; class: Arachnida; order: Prostigmata; family: Eriophyidae; genus: Cecidophyopsis; **Location:** country: Saudi Arabia; stateProvince: Abha; verbatimCoordinates: 20°04'0.91"N 41°20'1.25"E; decimalLatitude: 41.3354172; decimalLongitude: 20.068183; georeferenceProtocol: GPS; **Identification:** identifiedBy: Eid Muhammad Khan; dateIdentified: 2021; **Event:** samplingProtocol: Leaves collection; eventDate: 15/09/2021; habitat: Conocarpus erectus ; **Record Level:** language: en; collectionCode: Mites; basisOfRecord: Slide Mounted Specimen
- **Type status:** Other material. **Occurrence:** catalogNumber: KSMAAS-21-Eri-Cec-07; recordedBy: E.M. Khan; individualCount: 1; sex: Female; lifeStage: adult; occurrenceID: CD99B5A0-80DB-5469-AD1D-80ECCE6B72F4; **Taxon:** scientificName: Cecidophyopsis vermiformis ; kingdom: Animalia; phylum: Arthropoda; class: Arachnida; order: Prostigmata; family: Eriophyidae; genus: Cecidophyopsis; **Location:** country: Saudi Arabia; stateProvince: Abha; verbatimCoordinates: 20°04'0.91"N 41°20'1.25"E; decimalLatitude: 41.3354172; decimalLongitude: 20.068183; georeferenceProtocol: GPS; **Identification:** identifiedBy: Eid Muhammad Khan; dateIdentified: 2021; **Event:** samplingProtocol: Leaves collection; eventDate: 15/09/2021; habitat: Conocarpus erectus ; **Record Level:** language: en; collectionCode: Mites; basisOfRecord: Slide Mounted Specimen
- **Type status:** Other material. **Occurrence:** catalogNumber: KSMAAS-21-Eri-Cec-08; recordedBy: E.M. Khan; individualCount: 1; sex: Female; lifeStage: adult; occurrenceID: BFD23E5B-B3C3-500A-B475-557BC7A5D1A9; **Taxon:** scientificName: Cecidophyopsis vermiformis ; kingdom: Animalia; phylum: Arthropoda; class: Arachnida; order: Prostigmata; family: Eriophyidae; genus: Cecidophyopsis; **Location:** country: Saudi Arabia; stateProvince: Abha; verbatimCoordinates: 20°04'0.91"N 41°20'1.25"E; decimalLatitude: 41.3354172; decimalLongitude: 20.068183; georeferenceProtocol: GPS; **Identification:** identifiedBy: Eid Muhammad Khan; dateIdentified: 2021; **Event:** samplingProtocol: Leaves collection; eventDate: 15/09/2021; habitat: Conocarpus erectus ; **Record Level:** language: en; collectionCode: Mites; basisOfRecord: Slide Mounted Specimen
- **Type status:** Other material. **Occurrence:** catalogNumber: KSMAAS-21-Eri-Cec-09; recordedBy: E.M. Khan; individualCount: 1; sex: Female; lifeStage: adult; occurrenceID: 592D8758-481C-5E93-BC93-01D85A3C6A3A; **Taxon:** scientificName: Cecidophyopsis vermiformis ; kingdom: Animalia; phylum: Arthropoda; class: Arachnida; order: Prostigmata; family: Eriophyidae; genus: Cecidophyopsis; **Location:** country: Saudi Arabia; stateProvince: Abha; verbatimCoordinates: 20°04'0.91"N 41°20'1.25"E; decimalLatitude: 41.3354172; decimalLongitude: 20.068183; georeferenceProtocol: GPS; **Identification:** identifiedBy: Eid Muhammad Khan; dateIdentified: 2021; **Event:** samplingProtocol: Leaves collection; eventDate: 15/09/2021; habitat: Conocarpus erectus ; **Record Level:** language: en; collectionCode: Mites; basisOfRecord: Slide Mounted Specimen
- **Type status:** Other material. **Occurrence:** catalogNumber: KSMAAS-21-Eri-Cec-10; recordedBy: E.M. Khan; individualCount: 1; sex: Female; lifeStage: adult; occurrenceID: EC5DBB8D-7214-507C-B711-BEF92AE7A95F; **Taxon:** scientificName: Cecidophyopsis vermiformis ; kingdom: Animalia; phylum: Arthropoda; class: Arachnida; order: Prostigmata; family: Eriophyidae; genus: Cecidophyopsis; **Location:** country: Saudi Arabia; stateProvince: Abha; verbatimCoordinates: 20°04'0.91"N 41°20'1.25"E; decimalLatitude: 41.3354172; decimalLongitude: 20.068183; georeferenceProtocol: GPS; **Identification:** identifiedBy: Eid Muhammad Khan; dateIdentified: 2021; **Event:** samplingProtocol: Leaves collection; eventDate: 15/09/2021; habitat: Conocarpus erectus ; **Record Level:** language: en; collectionCode: Mites; basisOfRecord: Slide Mounted Specimen

#### Distribution

**Figure 3.**
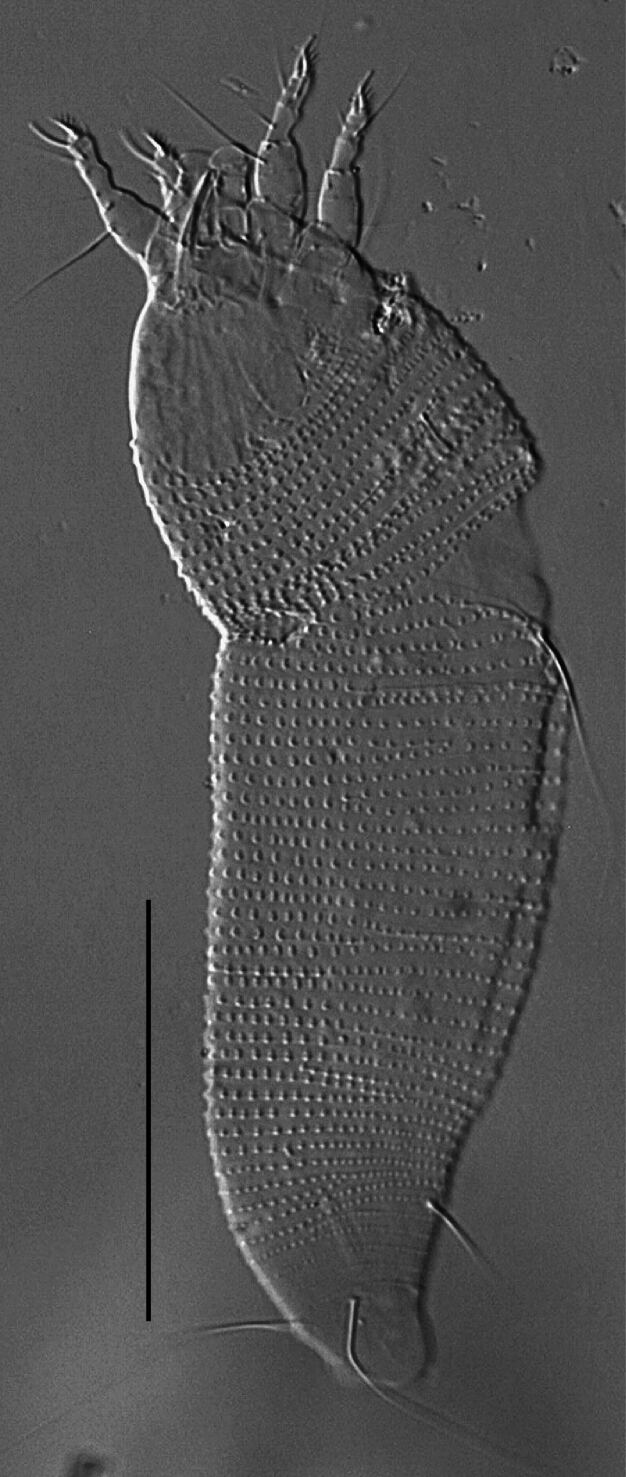
*Cecidophyopsis vermiformis* (Nalepa), female, dorso-lateral view. Scale bar 50 µm.

Armenia, Bulgaria, Finland, France, Germany, Hungary, Italy, Poland, Turkiye, USA (Amrine and de Lillo unpublished eriophyoid database) and Saudi Arabia (present study) (Fig. 3).

#### Ecology

**Figure 4.**
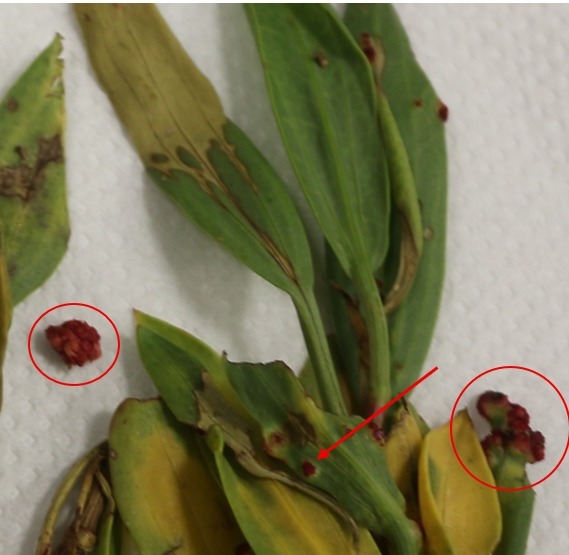
Pink gall on *Conocorpus erectus* by *Cecidophyopsis vermiformis* (Nalepa).

**Relation to the host plant**. Causes large pink galls (Fig. 4).

### Neserella capreifoliae

Meyer & Ueckermann

E932F835-088E-57D7-85FD-C8CF380D5DC4

#### Materials

- **Type status:** Other material. **Occurrence:** catalogNumber: KSMAAS-22-Eri-Nes-01; recordedBy: E.M. Khan; individualCount: 1; sex: Female; lifeStage: adult; occurrenceID: D6FB268E-1B75-5F4A-87C1-B952F7E174B2; **Taxon:** scientificName: Neserella capreifoliae; kingdom: Animalia; phylum: Arthropoda; class: Arachnida; order: Prostigmata; family: Eriophyidae; genus: Neserella; **Location:** country: Saudi Arabia; stateProvince: Jazan; verbatimCoordinates: 17°15'23.4"N 43°6'20.519"E; decimalLatitude: 17.2565; decimalLongitude: 43.105694; georeferenceProtocol: GPS; **Identification:** identifiedBy: Eid Muhammad Khan; dateIdentified: 2022; **Event:** samplingProtocol: Leaves collection; eventDate: 16/05/2022; habitat: Ficus carica ; **Record Level:** language: en; collectionCode: Mites; basisOfRecord: Slide Mounted Specimen
- **Type status:** Other material. **Occurrence:** catalogNumber: KSMAAS-22-Eri-Nes-02; recordedBy: E.M. Khan; individualCount: 1; sex: Female; lifeStage: adult; occurrenceID: AD9B8834-D8DC-54DF-958D-931FBC8E9838; **Taxon:** scientificName: Neserella capreifoliae; kingdom: Animalia; phylum: Arthropoda; class: Arachnida; order: Prostigmata; family: Eriophyidae; genus: Neserella; **Location:** country: Saudi Arabia; stateProvince: Jazan; verbatimCoordinates: 17°15'23.4"N 43°6'20.519"E; decimalLatitude: 17.2565; decimalLongitude: 43.105694; georeferenceProtocol: GPS; **Identification:** identifiedBy: Eid Muhammad Khan; dateIdentified: 2022; **Event:** samplingProtocol: Leaves collection; eventDate: 16/05/2022; habitat: Ficus carica ; **Record Level:** language: en; collectionCode: Mites; basisOfRecord: Slide Mounted Specimen
- **Type status:** Other material. **Occurrence:** catalogNumber: KSMAAS-22-Eri-Nes-03; recordedBy: E.M. Khan; individualCount: 1; sex: Female; lifeStage: adult; occurrenceID: 32F0EDB7-11E9-5822-BA3D-1F308BE577C6; **Taxon:** scientificName: Neserella capreifoliae; kingdom: Animalia; phylum: Arthropoda; class: Arachnida; order: Prostigmata; family: Eriophyidae; genus: Neserella; **Location:** country: Saudi Arabia; stateProvince: Jazan; verbatimCoordinates: 17°15'23.4"N 43°6'20.519"E; decimalLatitude: 17.2565; decimalLongitude: 43.105694; georeferenceProtocol: GPS; **Identification:** identifiedBy: Eid Muhammad Khan; dateIdentified: 2022; **Event:** samplingProtocol: Leaves collection; eventDate: 16/05/2022; habitat: Ficus carica ; **Record Level:** language: en; collectionCode: Mites; basisOfRecord: Slide Mounted Specimen
- **Type status:** Other material. **Occurrence:** catalogNumber: KSMAAS-22-Eri-Nes-04; recordedBy: E.M. Khan; individualCount: 1; sex: Female; lifeStage: adult; occurrenceID: 4AD32568-E49A-5897-9BAB-CCACE31421A7; **Taxon:** scientificName: Neserella capreifoliae; kingdom: Animalia; phylum: Arthropoda; class: Arachnida; order: Prostigmata; family: Eriophyidae; genus: Neserella; **Location:** country: Saudi Arabia; stateProvince: Jazan; verbatimCoordinates: 17°15'23.4"N 43°6'20.519"E; decimalLatitude: 17.2565; decimalLongitude: 43.105694; georeferenceProtocol: GPS; **Identification:** identifiedBy: Eid Muhammad Khan; dateIdentified: 2022; **Event:** samplingProtocol: Leaves collection; eventDate: 16/05/2022; habitat: Ficus carica ; **Record Level:** language: en; collectionCode: Mites; basisOfRecord: Slide Mounted Specimen
- **Type status:** Other material. **Occurrence:** catalogNumber: KSMAAS-22-Eri-Nes-05; recordedBy: E.M. Khan; individualCount: 1; sex: Female; lifeStage: adult; occurrenceID: 16F8FA61-5BC1-54CE-A0AF-915D5601511D; **Taxon:** scientificName: Neserella capreifoliae; kingdom: Animalia; phylum: Arthropoda; class: Arachnida; order: Prostigmata; family: Eriophyidae; genus: Neserella; **Location:** country: Saudi Arabia; stateProvince: Tabuk; verbatimCoordinates: 28°20'18.7"N 36°22'46.012"E; decimalLatitude: 28.338528; decimalLongitude: 36.379448; georeferenceProtocol: GPS; **Identification:** identifiedBy: Eid Muhammad Khan; dateIdentified: 2022; **Event:** samplingProtocol: Leaves collection; eventDate: 20/09/2022; habitat: Ficus carica ; **Record Level:** language: en; collectionCode: Mites; basisOfRecord: Slide Mounted Specimen
- **Type status:** Other material. **Occurrence:** catalogNumber: KSMAAS-22-Eri-Nes-06; recordedBy: E.M. Khan; individualCount: 1; sex: Female; lifeStage: adult; occurrenceID: B6C854B1-CA41-5047-8D46-807BC618291D; **Taxon:** scientificName: Neserella capreifoliae; kingdom: Animalia; phylum: Arthropoda; class: Arachnida; order: Prostigmata; family: Eriophyidae; genus: Neserella; **Location:** country: Saudi Arabia; stateProvince: Tabuk; verbatimCoordinates: 28°20'18.7"N 36°22'46.012"E; decimalLatitude: 28.338528; decimalLongitude: 36.379448; georeferenceProtocol: GPS; **Identification:** identifiedBy: Eid Muhammad Khan; dateIdentified: 2022; **Event:** samplingProtocol: Leaves collection; eventDate: 20/09/2022; habitat: Ficus carica ; **Record Level:** language: en; collectionCode: Mites; basisOfRecord: Slide Mounted Specimen
- **Type status:** Other material. **Occurrence:** catalogNumber: KSMAAS-22-Eri-Nes-07; recordedBy: E.M. Khan; individualCount: 1; sex: Female; lifeStage: adult; occurrenceID: 5BCF7326-0F5F-546D-8A68-6D1C8F47B28C; **Taxon:** scientificName: Neserella capreifoliae; kingdom: Animalia; phylum: Arthropoda; class: Arachnida; order: Prostigmata; family: Eriophyidae; genus: Neserella; **Location:** country: Saudi Arabia; stateProvince: Tabuk; verbatimCoordinates: 28°20'18.7"N 36°22'46.012"E; decimalLatitude: 28.338528; decimalLongitude: 36.379448; georeferenceProtocol: GPS; **Identification:** identifiedBy: Eid Muhammad Khan; dateIdentified: 2022; **Event:** samplingProtocol: Leaves collection; eventDate: 20/09/2022; habitat: Ficus carica ; **Record Level:** language: en; collectionCode: Mites; basisOfRecord: Slide Mounted Specimen
- **Type status:** Other material. **Occurrence:** catalogNumber: KSMAAS-22-Eri-Nes-08; recordedBy: E.M. Khan; individualCount: 1; sex: Female; lifeStage: adult; occurrenceID: 8B9F4330-F869-5C8F-9520-F83A51A4F466; **Taxon:** scientificName: Neserella capreifoliae; kingdom: Animalia; phylum: Arthropoda; class: Arachnida; order: Prostigmata; family: Eriophyidae; genus: Neserella; **Location:** country: Saudi Arabia; stateProvince: Tabuk; verbatimCoordinates: 28°20'18.7"N 36°22'46.012"E; decimalLatitude: 28.338528; decimalLongitude: 36.379448; georeferenceProtocol: GPS; **Identification:** identifiedBy: Eid Muhammad Khan; dateIdentified: 2022; **Event:** samplingProtocol: Leaves collection; eventDate: 20/09/2022; habitat: Ficus carica ; **Record Level:** language: en; collectionCode: Mites; basisOfRecord: Slide Mounted Specimen
- **Type status:** Other material. **Occurrence:** catalogNumber: KSMAAS-22-Eri-Nes-09; recordedBy: E.M. Khan; individualCount: 1; sex: Female; lifeStage: adult; occurrenceID: 66CF5575-1517-58D7-BD51-C51213D0DED8; **Taxon:** scientificName: Neserella capreifoliae; kingdom: Animalia; phylum: Arthropoda; class: Arachnida; order: Prostigmata; family: Eriophyidae; genus: Neserella; **Location:** country: Saudi Arabia; stateProvince: Tabuk; verbatimCoordinates: 28°20'18.7"N 36°22'46.012"E; decimalLatitude: 28.338528; decimalLongitude: 36.379448; georeferenceProtocol: GPS; **Identification:** identifiedBy: Eid Muhammad Khan; dateIdentified: 2022; **Event:** samplingProtocol: Leaves collection; eventDate: 20/09/2022; habitat: Ficus carica ; **Record Level:** language: en; collectionCode: Mites; basisOfRecord: Slide Mounted Specimen
- **Type status:** Other material. **Occurrence:** catalogNumber: KSMAAS-22-Eri-Nes-10; recordedBy: E.M. Khan; individualCount: 1; sex: Female; lifeStage: adult; occurrenceID: 9D0FE72B-AEDD-5093-A96B-770F516F4AA5; **Taxon:** scientificName: Neserella capreifoliae; kingdom: Animalia; phylum: Arthropoda; class: Arachnida; order: Prostigmata; family: Eriophyidae; genus: Neserella; **Location:** country: Saudi Arabia; stateProvince: Tabuk; verbatimCoordinates: 28°20'18.7"N 36°22'46.012"E; decimalLatitude: 28.338528; decimalLongitude: 36.379448; georeferenceProtocol: GPS; **Identification:** identifiedBy: Eid Muhammad Khan; dateIdentified: 2022; **Event:** samplingProtocol: Leaves collection; eventDate: 20/09/2022; habitat: Ficus carica ; **Record Level:** language: en; collectionCode: Mites; basisOfRecord: Slide Mounted Specimen

#### Distribution

**Figure 5.**
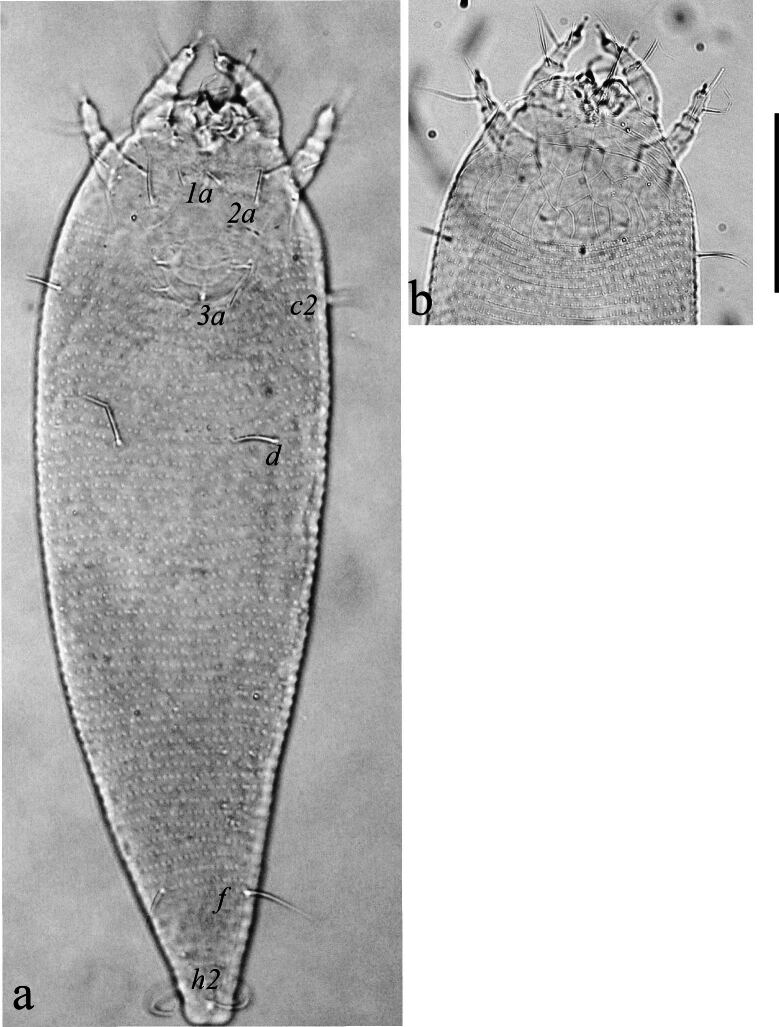
*Neserella capreifoliae* Meyer & Ueckermann Female; **a** Ventral view; **b** Prodorsal shield, Scale bar 50 µm.

South Africa (Meyer and Ueckermann 1989), Thailand (Chandrapatya et al. 2016), Egypt (Elhalawany et al. 2022) and Saudi Arabia (present study) (Fig. 5).

#### Ecology

**Relation to the host plant**. Vagrant on the abaxial surface of the leaf. No apparent symptoms to the host foliage were observed.

## Identification Keys

### Key to species of the genus *Cosetacus* Keifer (seven species, including new species)

**Table:** 

1	Setae *h_1_* present	2
–	Setae *h_1_* absent	3
2	Coxisternal plates smooth	*C. prosteti* Pandit & Chakrabarti
–	Coxisternal plates with granules and short lines	*C. javanicus***sp. nov**.
3	Empodium divided	*C. sharadi* Menon, Joshi & Ramamurthy
–	Empodium entire	4
4	Empodium 3–rayed	*C. mamangi* Han & Zhang
–	Empodium more than 3-rayed	5
5	Empodium 4 to 5–rayed	6
–	Empodium 6–rayed	*C. camelliae* (Keifer)
6	Coxisternal plates smooth, prodorsal shield with median and admedian faint lines	*C. citrifolis* Das & Chakrabarti
–	Coxisternal plates with granules and short lines, prodorsal shield with number of closely set discontinued lines	*C. eupatori* Chakrabarti & Pandit

## Supplementary Material

XML Treatment for Cosetacus javanicus

XML Treatment for Cecidophyopsis vermiformis

XML Treatment for Neserella capreifoliae
